# Consistency of c‐Met protein overexpression over time in patients with non‐squamous non‐small cell lung cancer

**DOI:** 10.1111/his.70168

**Published:** 2026-06-09

**Authors:** Alexis B Cortot, Romain Dubois, Valérie Grégoire, Jean‐Baptiste Gibier, Julien Labreuche, Virginie Deprez, Déborah Dubrulle, Thomas Chretien, Eric Wasielewski, Hélène Behal, Dai Feng, Amber Lind, Peter Ansell, Raphaële Thiébaut‐Millot, Carlos Hader

**Affiliations:** ^1^ CNRS, Inserm, Institut Pasteur de Lille, UMR9020‐UMR1277‐Canther‐Cancer Heterogeneity, Plasticity and Resistance to Therapies, Univ. Lille CHU Lille Lille France; ^2^ Institute of Pathology CHU Lille Lille France; ^3^ Department of Biostatistics CHU Lille Lille France; ^4^ Department of Thoracic Oncology CHU Lille Lille France; ^5^ AbbVie Inc North Chicago Illinois USA

**Keywords:** c‐Met protein overexpression, *EGFR* mutation, immunohistochemistry, NSCLC, status consistency

## Abstract

**Aims:**

The c‐Met signalling pathway is dysregulated in non‐small cell lung cancer (NSCLC), with c‐Met protein overexpression emerging as a potentially actionable biomarker given the development of c‐Met‐targeted antibody‐drug conjugates. This retrospective study assessed the consistency of c‐Met protein overexpression over time in patients with non‐squamous NSCLC and two or more longitudinal biopsy samples (one before treatment, one post‐treatment).

**Methods and results:**

Samples from 160 patients were analysed for c‐Met protein expression. The concordance rate for c‐Met protein overexpression between samples was 81.3%, indicating most patients had consistent expression status. Patients with an *EGFR* mutation had a lower concordance rate (65.8%). Fourteen patients with high discordance (negative to positive) between sampling time points showed a high frequency of oncogenic driver alterations, hence often received targeted therapy before the second sample and had high percentages of cells at 2+ intensity in c‐Met immunohistochemistry before treatment.

**Conclusions:**

These results indicate c‐Met protein overexpression can be assessed before or after treatment since most patients maintain consistent c‐Met status. As targeted therapies may elevate c‐Met overexpression over time, retesting may be necessary in those with an oncogenic driver alteration initially diagnosed as c‐Met negative.

## Graphical abstract

c‐Met protein overexpression can be assessed before or after treatment in patients with non‐squamous NSCLC since most patients maintain a consistent c‐Met status. However, as targeted therapies may elevate c‐Met overexpression, retesting may be necessary for subsets of patients.

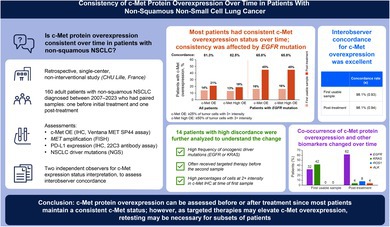

**Abbreviations**
CHU Lille: Centre Hospitalier Universitaire de Lille
FFPE: formalin‐fixed paraffin‐embedded
IHC: immunohistochemistry
NGS: next‐generation sequencing
NSCLC: non‐small cell lung cancer
Teliso‐V: telisotuzumab vedotin
Temab‐A: telisotuzumab adizutecan

## Introduction

Lung cancer has the highest mortality among malignant tumours. The majority of lung cancers (~85%) are non‐small cell lung cancer (NSCLC), and these are mainly non‐squamous in nature.^1^ Non‐squamous NSCLC is a heterogeneous disease with various potential driver gene alterations, including *EGFR*, *ALK*, *KRAS* and *ROS1* alterations.^2^ Current standard of care for first‐line treatment of advanced non‐squamous NSCLC depends on the presence/absence of these oncogenic drivers. In the first‐line setting, patients with oncogenic driver alterations generally receive targeted therapy.^3^, ^4^ In the absence of a driver gene alteration, the most common first‐line strategy is immunotherapy targeting programmed cell death protein 1 (PD‐1) or its ligand (PD‐L1) with or without chemotherapy, depending on PD‐L1 expression levels.^3^, ^5^ After disease progression, treatment options are mainly restricted to chemotherapy, which often has limited benefit and can lead to systemic toxicity.^6^ Biomarker‐directed therapy for second‐line treatment of non‐squamous NSCLC has the potential to greatly improve outcomes.

The tyrosine kinase receptor c‐Met (also known as MET) protein is encoded by the *MET* proto‐oncogene. The c‐Met signalling pathway is dysregulated in various cancers, including NSCLC. The most common MET alterations are c‐Met protein overexpression, *MET* amplification and *MET* exon 14 skipping mutations. These alterations are associated with carcinogenesis, proliferation, treatment resistance and poor clinical outcomes.^7^, ^8^, ^9^ c‐Met protein overexpression is the most frequent MET alteration, with a recent study reporting a prevalence of 24% in patients with *EGFR*‐wildtype non‐squamous NSCLC when defining overexpression as an immunohistochemistry (IHC) result of ≥25% tumour cells with 3+ intensity,^10^ and is emerging as a potentially actionable biomarker and therapeutic target.^11^

Several c‐Met protein–directed therapies are in development or approved, including the antibody‐drug conjugates telisotuzumab vedotin (Teliso‐V), telisotuzumab adizutecan (Temab‐A; ABBV‐400), TR1801 and the bispecific antibody MCLA‐129.^12^, ^13^, ^14^, ^15^, ^16^ Teliso‐V is composed of a humanized monoclonal antibody (telisotuzumab) conjugated to the cytotoxic microtubule inhibitor monomethyl auristatin E via a cleavable valine‐citrulline linker.^17^ Teliso‐V monotherapy was investigated in patients with previously treated, c‐Met protein–overexpressing, *EGFR*‐wildtype advanced/metastatic non‐squamous NSCLC in the phase 2, multicentre, non‐randomized LUMINOSITY trial. The overall response rate among 161 patients was 29% and this was enriched to 35% among patients with c‐Met high overexpressing tumours, defined as ≥50% of tumour cells with 3+ intensity.^10^ On the basis of these results, Teliso‐V was approved by the US Food and Drug Administration for treatment of patients with locally advanced/metastatic, non‐squamous NSCLC and high c‐Met protein overexpression who received prior systemic therapy.^18^

Information on the consistency of c‐Met protein overexpression over time or across lines of therapy is limited. Consequently, uncertainty remains as to when c‐Met protein expression should be assessed and whether repeat biopsy is necessary. Improved understanding of this topic will be crucial to inform the testing strategy for c‐Met–directed therapy. Herein, we present results from a retrospective study that investigated the consistency of c‐Met protein overexpression over time in patients with non‐squamous NSCLC. In addition, results on the association of c‐Met protein overexpression with other biomarkers in NSCLC and interobserver agreement on c‐Met protein overexpression status are presented.

## Materials and Methods

### Study Design

This retrospective, single‐centre, non‐interventional study included adult (≥18 years) patients with non‐squamous NSCLC who were diagnosed between 2007 and 2023 and received treatment at Centre Hospitalier Universitaire de Lille (CHU Lille). Patients were required to have at least two longitudinal formalin‐fixed paraffin‐embedded (FFPE) samples available, one taken prior to initial treatment and one taken post‐treatment. The only treatment allowed prior to collection of the first sample was surgery (prior surgery was not part of the inclusion criteria). If there was insufficient or poor‐quality tissue and a lack of results from routine clinical practice for c‐Met IHC, patients were excluded.

Tumour tissue was collected as part of the routine practice in CHU Lille from patients who underwent biopsy and/or resection procedures. Clinical data, treatment history and follow‐up data were collected from medical records. A detailed statement of processing of personal data was made to the CHU Lille Data Protection Officer (reference DEC23‐057). Per general data protection regulation, patients were informed using medical letters (mention of possible data reuse and possibility of opposition) and/or a specific information letter about this study. Oppositions were recorded in medical files and the Hospital Information System. A Privacy Impact Assessment was performed before any data treatment and all data were de‐identified before collection, storage and treatment.

### Assessments

Freshly cut slices from longitudinal FFPE samples were analysed for c‐Met protein expression, *MET* amplification and PD‐L1 expression (Assessment methods in Supplementary materials). c‐Met protein overexpression was defined as ≥25% of tumour cells with 3+ membrane staining; c‐Met high overexpression was defined as ≥50% of tumour cells with 3+ membrane staining (same definitions as in the LUMINOSITY trial^10^). When quality or quantity of samples was insufficient, results from routine clinical practice were considered for data collection, if available. *MET* exon 14 skipping mutation status and presence of NSCLC driver alterations were determined through next‐generation sequencing (NGS). For NGS, results from routine clinical practice were first considered for data collection and, if this was unavailable, NGS testing was performed. The priority order of analyses based on sufficient quality and quantity was (1) c‐Met IHC, (2) *MET* amplification, (3) PD‐L1 expression and (4) NGS. Statistical methods are in Supplementary materials.

## Results

### Patients

A total of 160 patients were included in the analysis (Table 1). The median patient age was 66 years (range 36–88) and most patients had adenocarcinoma (94%), metastatic disease (96%) and stage IV disease (64%) at diagnosis. At time of second sampling, the median number of prior treatments was one (range 1–8).

**Table 1:** Patient demographics and characteristics at diagnosis

	Overall (*N* = 160)
Median age, years (range)	66 (36–88)
Sex, *n* (%)	
Male	102 (64)
Female	58 (36)
Diagnosis [histology], *n* (%)	
Adenocarcinoma	151 (94)
Large cell carcinoma	1 (1)
Sarcomatoid carcinoma	1 (1)
Not otherwise specified	6 (4)
Other	1 (1)
Metastasis, *n* (%)	154 (96)
Smoking status, *n* (%)^a^	
Current	60 (41)
Former	54 (36)
Never	34 (23)
ECOG performance status, *n* (%)^b^	
0	62 (50)
1	57 (46)
2	6 (5)
Stage at diagnosis, *n* (%)^c^	
I	15 (10)
II	9 (6)
III	30 (20)
IV	98 (64)
Patients who received surgery prior to first usable sample, *n* (%)	4 (3)
Median number of treatment lines received at second sample (range)^c^	1 (1–8)
Type of systemic treatment received between first and second samples, *n* (%)^c^	
Chemotherapy	117 (74)
Immunotherapy	37 (23)
Targeted therapy	46 (29)

ECOG, Eastern Cooperative Oncology Group.

^a^ Assessed in 148 patients.

^b^ Assessed in 125 patients.

^c^ Assessed in 158 patients.

### Tumour Sampling Results

Median time from diagnosis to first sampling was 0 (range 0–94) months, as for most patients the first sample also served as the diagnostic sample. In a few cases (three patients), the diagnostic sample was too old to be used. In these rare cases, patients were included if a new sample was available at relapse and no treatment had been administered between the diagnostic sample and the relapse. For consistency, the date of the diagnostic sample was retained as the date of diagnosis for the three cases. Median time from diagnosis to post‐treatment sample was 14.4 (interquartile range 6.8–24.6) months and from first usable sample (hereafter referred to as ‘first sample’ for simplicity [except for in figures/tables]) to post‐treatment sample was 15.1 (interquartile range 7.1–25.6) months. The most common types of samples collected for the first sample were bronchial biopsy (26%), metastasectomy (19%) or endoscopic ultrasound aspiration (18%). For the post‐treatment sample, the most common types were metastasectomy (28%) or computed tomography‐guided lung biopsy (16%). Site of sampling was more frequently primary (52%) for the first sample and metastasis (71%) for the post‐treatment sample (Table 2). Out of the 160 patients included, routine clinical practice results were used for 25 patients. For 23 patients, this was needed for only one sample, and for two patients, this was needed for both samples.

**Table 2:** Characteristics and findings of tumour samples

*n*/*N* (%)^a^	First usable sample	Post‐treatment sample
*Total population*		
Sampling origin		
Histologic	122/160 (76)	117/160 (73)
Cytologic	38/160 (24)	43/160 (27)
Site of sampling		
Primary	83/159 (52)	34/159 (21)
Metastasis	52/159 (33)	113/159 (71)
Mediastinal lymph node	24/159 (15)	12/159 (8)
c‐Met IHC		
*Overexpression (≥25% tumour cells with 3+ intensity)*	*23/159 (14)*	*33/159 (21)*
*High overexpression (≥50% of tumour cells with 3+ intensity)*	*21/159 (13)*	*31/159 (19)*
*MET* amplification	7/148 (5)	11/128 (9)
PD‐L1 expression		
0%	97/154 (63)	87/151 (58)
1%–49%	33/154 (21)	33/151 (22)
≥50%	24/154 (16)	31/151 (21)
Genomic alterations^b^		
*EGFR*	38/158 (24)	
*ALK*	12/158 (8)	
*MET*	5/158 (3)	
*ROS1*	2/158 (1)	
*KRAS*	53/158 (34)	
*NRAS*	4/158 (3)	
*PTEN*	2/158 (1)	
*TP53*	88/158 (56)	
*PIK3CA*	3/158 (2)	
*c‐Met protein–overexpressing population*		
*MET* amplification	2/16 (13)	7/30 (23)
PD‐L1 expression		
0%	7/23 (30)	9/30 (30)
1%–49%	7/23 (30)	9/30 (30)
≥50%	9/23 (39)	12/30 (40)
Genomic alterations		
*EGFR*	6/19 (32)	16/26 (62)
*ALK*	0	1/26 (4)
*ROS1*	0	2/26 (8)
*KRAS*	8/19 (42)	1/26 (4)

ALK, anaplastic lymphoma kinase; *EGFR*, epidermal growth factor receptor; IHC, immunohistochemistry; *KRAS*, Kirsten rat sarcoma viral oncogene homologue; *NRAS*, neuroblastoma RAS viral oncogene homologue; PD‐L1, programmed cell death protein 1 ligand 1; *PIK3CA*, phosphatidylinositol‐4,5‐bisphosphate 3‐kinase catalytic subunit alpha; PTEN, phosphatase and tensin homologue; *ROS1*, ROS proto‐oncogene 1, receptor tyrosine kinase; *TP53*, tumour protein p53.

^a^ Total *N* varied per analysis and sampling time points due to priority order testing.

^b^ Among patients with results for either the first usable or post‐treatment sample. For example, *EGFR* mutations (in either the first usable or post‐treatment sample) were detected in a total of 38 patients, while 119 patients tested negative for *EGFR* mutations in both samples or at one sampling time point with the test not performed for the other sample. For 2 patients, no results were available for either sampling time point.

*MET* amplification (Figure S1) was detected in seven (5%) patients in the first sample and 11 (9%) in the post‐treatment sample. The distribution of PD‐L1 expression was similar for both sampling time points across the total population. The most frequently occurring driver oncogene alterations were *EGFR* (*n* = 38/158, 24%) and *KRAS* (*n* = 53/158, 34%) (Table 2).

### Interobserver Concordance for c‐Met Protein Overexpression

**Figure 1:**
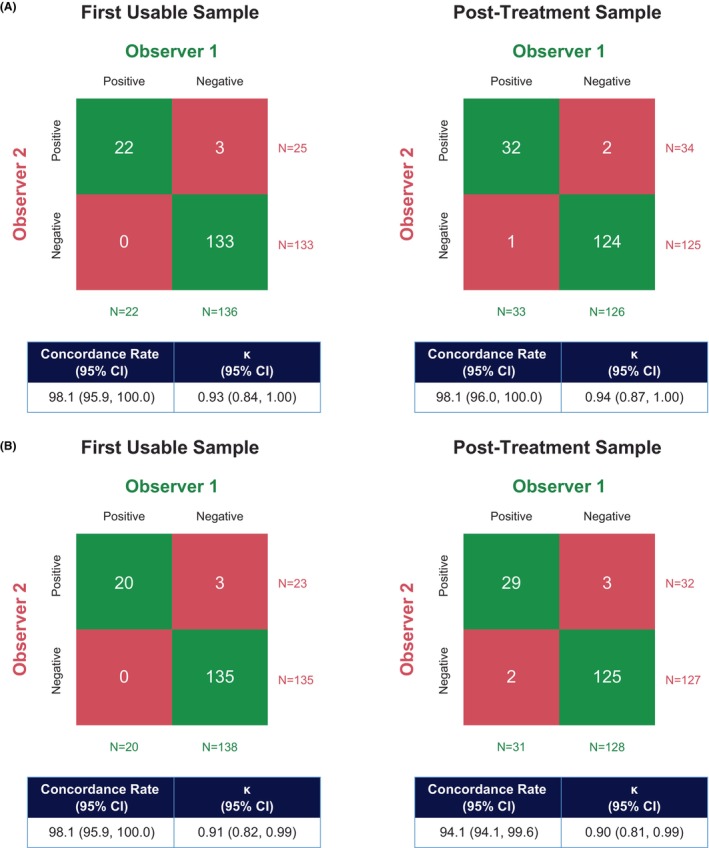
(**A**) Interobserver concordance for c‐Met protein overexpression in first usable and post‐treatment samples. (**B**) Interobserver concordance for c‐Met high overexpression in first usable and post‐treatment samples. CI, confidence interval.

c‐Met protein overexpression was assessed by two independent observers for each sample (Table S1). The interobserver concordance was excellent in both first and post‐treatment samples. For c‐Met protein overexpression in the first sample, the concordance rate was 98.1% (kappa coefficient: 0.93) (Figure 1A). In post‐treatment samples, the concordance rate was 98.1% (kappa coefficient: 0.94). Concordance results for c‐Met high overexpression were similar (Figure 1B). Considering these results, c‐Met protein overexpression data are presented for one observer only.

### c‐Met Protein Overexpression

**Figure 2:**
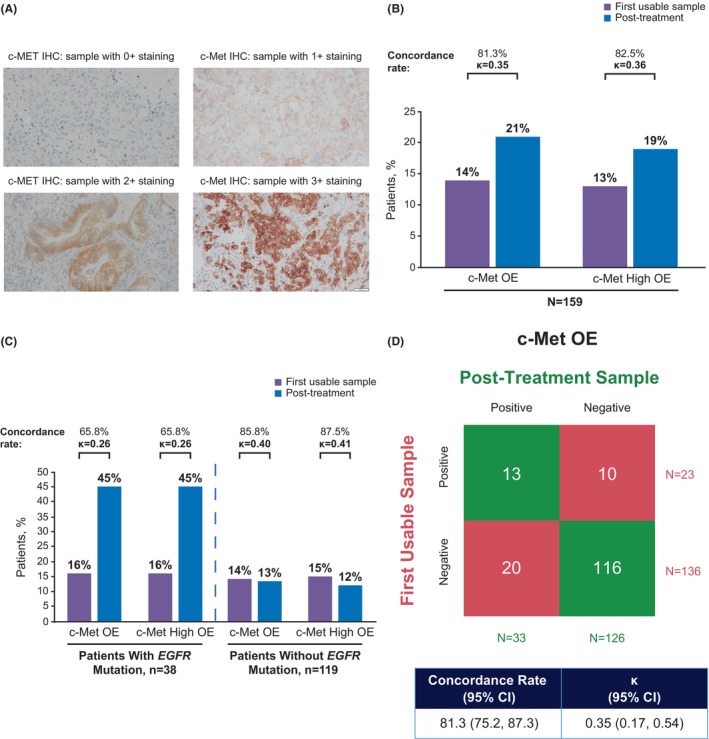
(**A**) Immunohistochemistry of c‐Met protein. (**B**) Concordance between first usable and post‐treatment samples for c‐Met and c‐Met high protein overexpression in the overall patient population. (**C**) Concordance between first usable and post‐treatment samples for c‐Met and c‐Met high protein overexpression in patients with and without *EGFR* mutations. All patients with *EGFR* mutations had c‐Met high overexpression. (**D**) Observer 1‐reported c‐Met protein overexpression status consistency and changes between the first usable sample and post‐treatment sample. CI, confidence interval; *EGFR*, epidermal growth factor receptor; IHC, immunohistochemistry; OE, overexpression.

c‐Met protein overexpression was detected in 23/159 (14%) first samples (Figure 2A). The majority of these patients had c‐Met high overexpression (21/159 [13%]). Overall, c‐Met protein overexpression occurred at a higher frequency in post‐treatment samples: 33/159 (21%) patients had c‐Met protein overexpression after treatment and 31/159 (19%) had c‐Met high overexpression (Figure 2B, Figure S2A). The concordance rate for c‐Met protein overexpression between first and post‐treatment samples was 81.3% (kappa coefficient: 0.35); for c‐Met high overexpression, concordance was 82.5% (kappa coefficient: 0.36).

Consistency in c‐Met protein overexpression was also assessed in patients with and without *EGFR* mutations. These mutations (in either sample) were detected in a total of 38 patients, while 119 patients tested negative for *EGFR* mutations in both samples or at one sampling time point with the test not performed for the other sample. The concordance between the first and post‐treatment samples for patients without an *EGFR* mutation was similar to the total population; however, a much lower concordance was seen for the subset with an *EGFR* mutation. In the first sample, c‐Met protein overexpression was detected in 6/38 (16%) patients vs in 17/38 (45%) in the post‐treatment sample (Figure 2C). The concordance rate was 65.8% (kappa coefficient: 0.26). All patients in this population had c‐Met high overexpression.

Small sample sizes limited comparison of c‐Met overexpression among different tissue sites; however, c‐Met overexpression was detected in 13/83 (16%) lung samples, 5/36 (14%) lymph node samples and 1/17 (6%) brain sample; c‐Met high expression was detected in 12/83 (15%), 4/36 (11%) and 1/17 (6%) lung, lymph node and brain samples, respectively.

A total of 129 (81%) patients had consistent c‐Met protein overexpression status in both the first and post‐treatment sample; 116 were negative in both samples and 13 were positive in both (Figure 2D, Figure S2B). For 30 patients, the c‐Met protein overexpression status changed between sampling time points; 20 were negative during the first sampling and positive in the post‐treatment sample, while 10 were positive during the first sampling and negative in the post‐treatment sample. There was no clear difference in time interval between sampling time points and c‐Met protein overexpression concordance (Table S2). Among the patients with a change in c‐Met status from negative to positive, 14 had high discordance between sampling time points (defined as an absolute change of >90% in cells with 3+ intensity between first and post‐treatment sample) and were studied in further detail to identify a potential cause for this change. Several defining features were identified for this population. Among the 14 patients, 12 had an evaluable driver oncogene status and these were all positive, either for *EGFR* (*n* = 10) or *KRAS* (*n* = 2). The majority of patients (12/14, 86%) had received targeted therapy prior to the post‐treatment sample. With regard to c‐Met protein expression, 12/14 (86%) patients had either 50%–99% (*n* = 5) or 100% (*n* = 7) positive cells at 2+ intensity at time of the first sample, indicating that the change from negative to positive was mainly driven by an increase in c‐Met staining intensity. Among patients with 50–99% 2+ cells, 3/5 had an *EGFR* mutation, and among patients with 100% 2+ cells, 6/7 had an *EGFR* mutation. No specific characteristic was identified for patients changing from positive to negative after treatment. This group did have a higher proportion of patients who had received more than one prior line of therapy (7/10, 70%) compared with patients who had no change in c‐Met status (50/128, 39%) or who changed from negative to positive (9/20, 45%) (Table S2).

To assess the effect of sample age on c‐Met protein overexpression status, samples were divided into those collected before 2014, those collected between 2014 and 2018, and those collected from January 2019 onwards. While some difference was seen between the oldest and most recent samples, with a higher rate of c‐Met protein overexpression in newer samples, the difference was limited and not expected to significantly impact the presented results (Table S3). It should also be noted that the number of samples collected before 2014 was limited (*n* = 8).

### Co‐Occurrence of c‐Met Protein Overexpression and Other Biomarkers

The co‐occurrence of c‐Met protein overexpression and other biomarkers changed over time. Both *MET* amplification and *EGFR* mutations were more common in post‐treatment samples of patients with c‐Met protein overexpression (Table 2). The frequency of *MET* amplification increased from 13% (*n* = 2/16) in the first samples to 23% (*n* = 7/30) in post‐treatment samples, while the frequency of *EGFR* mutations increased from 32% (*n* = 6/19) to 62% (*n* = 16/26), respectively. Conversely, *KRAS* mutations were more commonly detected in the first sample of patients with c‐Met protein overexpression, and their frequency decreased from 42% (*n* = 8/19) to 4% (*n* = 1/26) between sampling time points.

## Discussion

This retrospective study on paired samples from patients with non‐squamous NSCLC evaluated interobserver agreement on c‐Met protein overexpression by IHC, consistency of c‐Met protein overexpression over time and associations of c‐Met protein overexpression with other biomarkers of importance.

Interpretation of IHC results can be affected by interobserver variation, and c‐Met IHC evaluation has been considered challenging in past studies. A study among different centres found variation of 27%–83% in c‐Met protein expression positivity among 150 samples in an exploratory testing round. The consistency in scoring was greatly improved by training and communication between pathologists.^19^ The current study had excellent agreement for c‐Met IHC results between the two independent observers assessing the results, with a concordance rate of 98%. It is worth noting that these observers had been trained together and had the opportunity to cross‐check and harmonize their c‐Met IHC results in everyday practice. The consistency between the two observers allows for confidence about the reliability of the performed analysis. In addition, it indicates that the applied assay can have good real‐world reproducibility. Besides IHC interpretation, results can also be impacted by the varying methods to obtain, fix and process tissue samples used by different centres,^20^ and process standardization would help ensure quality testing. The current study used the same definition for c‐Met protein overexpression status as the phase 2 LUMINOSITY trial^10^ and the CHRYSALIS‐2 study,^21^ but there currently is no universally accepted definition for c‐Met protein overexpression.

This study was not designed to assess the prevalence of c‐Met protein overexpression, and prevalence numbers observed in the current study may not reflect the general population. c‐Met protein overexpression status remained consistent in 81% of patients over time. Although a large proportion of patients were negative for c‐Met protein overexpression, the Kappa coefficient metric incorporates both the degree of agreement in each direction and the number of observations in each group. In 20 patients (13%), the c‐Met status changed from negative in the initial sample to positive in the post‐treatment sample, and for 10 (6%) patients, the opposite change occurred. These results were similar to the LUMINOSITY study, where a limited number of paired tissue samples (*n* = 28) were investigated for changes in c‐Met protein expression. Of these, 75% were consistent over time, 18% changed from negative to positive, and 7% changed from positive to negative between biopsies.^10^

The co‐occurrence of c‐Met protein overexpression with other biomarkers changed over time. In patients with c‐Met protein overexpression, the frequency of *EGFR* mutations and *MET* amplification was higher in post‐treatment samples compared with initial samples, while the frequency of *KRAS* was higher in first samples. A study investigating the correlation between *EGFR* mutations and c‐Met protein overexpression in resected primary lung adenocarcinoma found that 69% of samples with high expression of c‐Met (defined in that study as ≥25% cell staining) had an *EGFR* mutation, similar to the 62% seen in post‐treatment samples in the current study. Prior lines of treatment were not defined for this study.^22^

Notably, in patients with *EGFR* mutations, the likelihood of a change of c‐Met status post‐treatment appeared to be greater than for patients without *EGFR* mutations. The frequency of c‐Met protein overexpression in this patient subset increased from 16% to 45% between sampling time points, compared with 14% to 13% in patients without *EGFR* mutations. When looking in detail at patients who had a clear change in c‐Met protein status from negative to positive after treatment, several key differences were seen compared with the total population. This subgroup had a high frequency of oncogenic driver alterations (*EGFR* or *KRAS*) and often received targeted therapy prior to the second sample and had high percentages of cells at 2+ intensity in c‐Met IHC at time of first sample. This suggests that patients with an oncogenic driver alteration who receive targeted therapy may become positive for c‐Met protein overexpression after treatment through an upregulation of c‐Met receptor production. This is in line with data showing that *MET* amplification is an established driver of treatment resistance in *EGFR*‐mutant NSCLC.^23^, ^24^ *MET* amplification can lead to treatment resistance in *EGFR*‐mutant NSCLC by activating signalling pathways downstream of *EGFR*, making *EGFR* signalling redundant.^25^, ^26^ In NSCLC with other oncogenic drivers, *MET* amplification has similarly been identified as a mechanism of treatment resistance, as seen for example in patients with *KRAS* G12C‐mutant cancer treated with adagrasib.^27^ These results indicate that while for most patients, samples taken prior to initial treatment could be used to evaluate c‐Met protein overexpression status, the possibility of a change in the patient's c‐Met protein overexpression over time needs to be considered when moving to future lines of treatment. Retesting may be needed to assess c‐Met protein overexpression after targeted therapy.

Given the retrospective nature of this study and the dependence on available samples and associated clinical data, several limitations should be acknowledged. The cohort included patients diagnosed between 2007 and 2023, resulting in variability in the duration of FFPE sample storage. Prolonged storage can increase the risk of underestimating tumour proportion scores as observed with PD‐L1 in NSCLC samples that were over 3‐year old.^28^ This could potentially result in false negatives. Intratumoral heterogeneity has also been shown for c‐Met expression^29^ and may also influence c‐Met IHC results. In adenocarcinoma of the lung, c‐Met expression was reported to be higher at sites of tumour invasion compared with central regions of the tumour.^29^

In conclusion, results from this study suggest that c‐Met protein overexpression status can be evaluated in biopsies taken before or after treatment, with archival tissue collected for diagnosis purposes being adequate for testing. Data also suggest that most patients maintain their c‐Met status, underscoring the possibility of testing for c‐Met at NSCLC diagnosis. Some patients may experience a c‐Met status change that requires retesting, particularly patients with oncogenic driver alterations who tested c‐Met negative at diagnosis and were treated with targeted therapies, as these agents could increase c‐Met protein overexpression over time.

## Author contributions

AC, RT, CH and PA conceived the study, interpreted results and wrote the manuscript. AC and EW led local project management. AC, EW and VD managed the regulatory agreement procedure. EW, VD, DD, TC managed the biobank, processed the biological samples and data capture. RD, VG and JBG led pathologist interpretation and provided pathological expertise. JL and HB led statistical analyses and DF and AL provided statistical advice and expertise. All authors critically reviewed, revised and approved the manuscript.

## Funding information

AbbVie funded this study and participated in the study design, research, analysis, data collection, interpretation of data and the review and approval of the publication. No honoraria or payments were made for authorship. Medical writing support was funded by AbbVie.

## Conflicts of interest

A. Cortot: Grants or contracts from Exeliom; consulting fees from Novartis, AbbVie, Roche, Exeliom, Pfizer, Janssen, Amgen, Takeda, AstraZeneca, MSD; payment or honoraria for lectures, presentations, speakers' bureaus, manuscript writing or educational events from Pfizer, Amgen, Takeda, Novartis, Roche, AstraZeneca, MSD, Janssen, BMS; support for attending meetings and/or travel from Roche, MSD, Novartis, Pfizer, AstraZeneca, Amgen, BMS; participation on a data safety monitoring board or advisory board from InhaTarget, Merck. R. Dubois, V. Grégoire, J. Gibier, J. Labreuche, V. Deprez, D. Dubrulle, T. Chretien, H. Behal: Nothing to disclose. E. Wasielewski: Expert boards, Takeda. D. Feng, A. Lind, P. Ansell, R. Thiébaut‐Millot, C. Hader: Employees of AbbVie and may own stock.

## Supporting information


**Data S1** Supplementary methods.
**Table S1**. c‐Met protein expression.
**Table S2**. Patients with and without c‐Met protein overexpression concordance.
**Table S3**. c‐Met protein overexpression by sample age.
**Figure S1**. *MET* amplification visualized using fluorescence in situ hybridization.
**Figure S2**. c‐Met protein overexpression data per observer 2.

## Data Availability

Research data are not shared.
