# Supplementary material for: Consistency of c‐Met protein overexpression over time in patients with non‐squamous non‐small cell lung cancer
Source: Histopathology. 2026 Jun 9;89(3):456–66. doi: 10.1111/his.70168 (PMC13441392; doi:10.1111/his.70168)
Supplement: Supplementary file 1 [file HIS-89-456-s001.docx]

# Consistency of c-Met protein overexpression over time in patients with non-squamous non-small cell lung cancer

**Authors:** Alexis B. Cortot^1^, Romain Dubois^2^, Valérie Grégoire^2^, Jean-Baptiste Gibier^2^, Julien Labreuche^3^, Virginie Deprez^2^, Déborah Dubrulle^4^, Thomas Chretien^2^, Eric Wasielewski^4^, Hélène Behal^3^, Dai Feng^5^, Amber Lind^5^, Peter Ansell^5^, Raphaële Thiébaut-Millot^5^, Carlos Hader^5^

**Affiliations:** ^1^CNRS, Inserm, Institut Pasteur de Lille, UMR9020-UMR1277-Canther-Cancer Heterogeneity, Plasticity and Resistance to Therapies, Univ. Lille, CHU Lille, Lille, France; ^2^CHU Lille, Institute of Pathology, Lille, France; ^3^CHU Lille, Department of Biostatistics, Lille, France; ^4^CHU Lille, Department of Thoracic Oncology, Lille, France; ^5^AbbVie Inc, North Chicago, IL, USA.

***Supplementary materials***

# Methods

*Assessments*

c-Met protein expression was assessed by IHC using the Ventana MET SP44 assay, via the ultraView detection kit (Roche Diagnostics). For quality control, tissue from lung adenocarcinoma with a strong c-Met expression score (3+ in 100% of tumor cells) was included on each slide. Two independent observers assessed all samples for c-Met expression status. c-Met protein overexpression was defined as ≥25% of tumor cells with 3+ staining; c-Met high overexpression was defined as ≥50% of tumor cells with 3+ staining. *MET* amplification was analyzed by fluorescence in situ hybridization using ZytoLight^®^ SPEC MET/CEN7 probes. The ratio of MET/CEP7 and copy number were assessed. *MET* amplification positive was defined as a MET/CEP7 ratio of ≥1.8 or the presence of clusters; *MET* amplification negative was defined as a MET/CEP7 ratio <1.8. PD-L1 expression was assessed by IHC using the 22C3 antibody assay (Dako, Inc.) and the tumor proportion score was determined and categorized in three levels: 0%, 1–49%, and ≥50%.

*Statistical analysis*

Quantitative variables were described as median (range) and categorical variables as numbers (percentages). Agreement between the two independent observers on c-Met protein overexpression status was assessed by reporting the percentage agreement and calculating Cohen’s kappa coefficient with its 95% confidence interval (CI). To measure and describe the consistency of c-Met protein overexpression status (as assessed by observer 1) before and after treatment, results were tabulated for both samples and Cohen’s kappa coefficient between the two evaluations was calculated with its 95% CI. A subgroup analysis was performed according to presence or absence of an *EGFR* mutation. The distribution of genomic and expression biomarkers detected by NGS are described in total and according to c-Met protein overexpression status. Data were analyzed using the SAS software package, release 9.4 (SAS Institute).

| **Supplementary Table S1. c-Met Protein Expression** | | |
| --- | --- | --- |
| **n/N (%)** | **First Usable Sample** | **Post-Treatment Sample** |
| Intensity 0 |  |  |
| *0%* | 81/159 (51) | 82/159 (52) |
| *1–24%* | 15/159 (9) | 16/159 (10) |
| *25–49%* | 9/159 (6) | 14/159 (9) |
| *50–99%* | 29/159 (18) | 19/159 (12) |
| *100%* | 25/159 (16) | 28/159 (18) |
| Intensity 1 |  |  |
| *0%* | 70/159 (44) | 79/159 (50) |
| *1–24%* | 28/159 (18) | 20/159 (13) |
| *25–49%* | 16/159 (10) | 14/159 (9) |
| *50–99%* | 33/159 (21) | 33/159 (21) |
| *100%* | 12/159 (8) | 13/159 (8) |
| Intensity 2 |  |  |
| *0%* | 68/159 (43) | 90/159 (57) |
| *1–24%* | 25/159 (16) | 18/159 (11) |
| *25–49%* | 23/159 (15) | 12/159 (8) |
| *50–99%* | 28/159 (18) | 26/159 (16) |
| *100%* | 15/159 (9) | 13/159 (8) |
| Intensity 3 |  |  |
| *0%* | 125/159 (79) | 118/159 (74) |
| *1–24%* | 11/159 (7) | 8/159 (5) |
| *25–49%* | 2/159 (1) | 2/159 (1) |
| *50–99%* | 13/159 (8) | 12/159 (8) |
| *100%* | 8/159 (5) | 19/159 (12) |

| **Supplementary Table S2. Patients With and Without c-Met Protein Overexpression Concordance** | | | |
| --- | --- | --- | --- |
|  | **Patients With No Change in c-Met Status (n=130)** | **Negative to Positive (n=20)** | **Positive to Negative (n=10)** |
| Time interval between samples, median, months (25th to 75th percentile) | 14.9 (6.8–24.9) | 11.8 (6.9–18.7) | 19.2 (10.8–26.6) |
| Number of prior lines of therapy, median (25th to 75th percentile)  >1 line, n/N (%) | 1 (1–2)  50/128 (39.1) | 1 (1–2)  9/20 (45.0) | 2 (1–3)  7/10 (70.0) |

| **Supplementary Table S3. c-MET protein overexpression by sample age** | | | |
| --- | --- | --- | --- |
|  | **Sample Collected** | | |
|  | **Before 2014** | **2014–2018** | **≥2019** |
| No c-Met protein overexpression, n (%)  total n=134 | 7 (88) | 80 (85) | 47 (83) |
| c-Met protein overexpression,  n (%)  total n=25 | 1 (13) | 14 (15) | 10 (18) |
| Total  N=159 | 8 | 94 | 57 |

**Supplementary Figure S1.** *MET* amplification visualized using fluorescence in situ hybridization.

**Supplementary Figure S2.** c-Met protein overexpression data per observer 2. (A) Concordance between first usable and post-treatment samples for c-Met protein overexpression and c-Met high overexpression in the overall patient population. (B) c-Met protein overexpression status consistency and changes between the first usable sample and post-treatment sample. CI, confidence interval; OE, overexpression.
